# Bridging the communication gap in foregut cancer: A qualitative exploration of patient and caregiver perspectives

**DOI:** 10.1016/j.soi.2026.100238

**Published:** 2026-03-06

**Authors:** Ioannis Liapis, Jaspinder S. Sanghera, Katie West, Rida Ahmad, Ahmed Abdalla, Larry Hearld, Martin J. Heslin, Smita Bhatia, Krista Mehari, Annabelle L. Fonseca

**Affiliations:** a University of Alabama at Birmingham, Department of Surgery, United States; b University of Alabama at Birmingham, Institute of Cancer Outcomes and Survivorship, United States; c Rice University, Department of Biosciences, United States; d University of South Alabama, Department of Surgery, United States; e University of South Alabama, Department of Interdisciplinary Oncology, United States; f Unversity of Alabama at Birmingham, Department of Health Services Administration, United States; g Vanderbilt University, Department of Psychology, United States

**Keywords:** Physician communication, Shared decision-making, Patient-centered communication, Southeastern U.S

## Abstract

**Background:**

Effective communication between patients, caregivers and the healthcare team is essential in foregut cancer care, where treatment often involves complex, high-risk decisions. Despite its importance, patient and caregiver perspectives on communication remain underexplored, particularly in underserved populations. This study aims to identify barriers and facilitators to communication from the perspectives of patients with foregut cancer and their caregivers.

**Study design:**

This qualitative study involved semi-structured interviews at a safety-net and tertiary care center in the Southeastern United States. Interviews were recorded, transcribed, and qualitatively analyzed using inductive thematic and content analysis with NVivo 14 software. Intercoder agreement exceeded 90%. The study adhered to consolidated criteria for reporting qualitative research (COREQ).

**Results:**

Forty-five individuals (30 patients, 15 caregivers) participated. Four key communication barriers were identified: (1) information overload and lack of clarity, (2) lack of clear expectation setting regarding prognosis and treatment impact, (3) lack of acknowledgement of patient or caregiver concerns, and (4) perceived inconsistencies in communication among providers. Five facilitators were described: (1) clear explanations using accessible language, (2) written or visual materials, (3) feeling heard, (4) opportunities to voice personal goals and concerns, and (5) presentation of treatment options and alternatives.

**Conclusion:**

Patients with foregut cancer and their caregivers face significant communication challenges that may impact trust, decision-making and satisfaction. Clear, empathetic and consistent communication that integrates patient goals and clarifies expectations is crucial to enhance quality of care and support shared decision-making. Insights gained will inform strategies to enhance communication and improve patient-centered care.

## Introduction

Foregut cancers i.e., cancers of the esophagus, stomach, pancreas, liver, and biliary system are characterized by high morbidity and mortality, with many patients presenting at an advanced stage and facing limited curative options.^1,2^ When resectable, treatment involves multimodal regimens combining challenging systemic therapies that cause deconditioning with complex surgical procedures that despite high morbidity rates, offer an overall low chance of cure.^3–6^ For patients and their families, this cancer journey is not only physically and financially taxing, but also cognitively and emotionally over-whelming.^7,8^

Foregut cancer management has become increasingly centralized, with care being delivered by multidisciplinary teams at tertiary, high-volume facilities. Patients and their caregivers communicate with a wide range of specialist physicians, nurses and ancillary staff.^9,10^ While this model has been shown to improve oncologic outcomes,^11^ it also presents challenges. Patients and caregivers navigate a complex, sometimes fragmented care landscape, interact with multiple providers, and absorb vast amounts of information to make decisions about high-risk and potentially high-reward treatments. However, the literature shows that patients and caregivers find it difficult to fully comprehend prognosis, treatment options and potential side-effects.^12^

Effective communication is essential in this context, to convey requisite information, develop trust, ensure that treatments align with patients’ values and goals, improve treatment compliance and clinical outcomes. Conversely, poor communication can be associated with decisional regret and decreased trust in the healthcare system.^13–17^ Despite this critical role, communication is not often prioritized, and patients’ and caregivers’ perspectives remain underexplored. This is particularly true in underserved populations, where low health literacy, socioeconomic disadvantage and cultural barriers may exacerbate the impact of inadequate communication.^18,19^ The objective of this study was to explore the communication experiences of patients with foregut cancer and their caregivers at a safety-net hospital. By identifying both barriers and facilitators, we sought to inform practical strategies to enhance patient-centered communication and improve cancer care delivery.

## Methods

### Study design

This study employed a qualitative design, using semi-structured interviews to explore barriers and facilitators to effective communication, using thematic analysis. The study protocols and associated materials were approved by the University of South Alabama (USA) Institutional Review Board (IRB-2003085–1). The study adhered to the consolidated criteria for reporting qualitative research (COREQ) guidelines (Supplementary 1).^20^

### Study setting and participant recruitment

The study was conducted at a tertiary care safety-net hospital in the Southeastern US that serves a diverse and underserved population across Alabama and the neighboring Gulf Coast states.^21^ Participants included English-speaking adults receiving treatment for foregut malignancies or the primary caregiver for such patients. Participants were recruited using purposive selection to capture experiences across the continuum of foregut cancer care. Sampling was informed by cancer type and stage, treatment phase, geographic location and self-reported barriers to cancer care. Treatment phases included patients with non-metastatic disease receiving neoadjuvant chemotherapy, those who completed neoadjuvant chemotherapy and surgical resection and were in the postoperative recovery or surveillance phases, as well as patients with metastatic disease who were receiving or had received systemic therapy alone. Recruitment occurred in outpatient oncology clinics by a non-clinical member of the research team.

### Interview guide development and participant interviews

Separate semi-structured interview guides were developed for patients and caregivers based on grounded theory methodology to explore their perspectives on barriers and facilitators to cancer care (Supplementary 2). The guides were pilot tested with two cancer patients and two caregivers to ensure clarity and relevance. Open-ended and probing questions were incorporated to maximize participant engagement and dialogue. The interview was designed to elicit perspectives on barriers and facilitators to navigating foregut cancer care; although communication was not the exclusive focus, it emerged consistently as a dominant theme across interviews. Interviews were conducted at a single time point for each participant; however, participants were interviewed at various stages of their care trajectory.

Participants were informed of the goals of the study, written consent was obtained, and interviews lasting 30–60 min were conducted by two female interviewers (KW, RA) in a private face-to-face setting. The interviewers were formally trained in qualitative interviewing, had no prior relationships with the participants and no known preconceived biases on the subject. Participants received compensation of $25 for their time. Field notes were made during the interviews to capture contextual details and nonverbal cues. No repeat interviews were deemed necessary. Interviews were carried out until thematic saturation was reached. Of the 35 patients and 16 caregivers that were approached, 5 patients and 1 caregiver declined participation, citing time constraints.

### Qualitative analysis

Interviews were audio recorded, professionally transcribed, verified for accuracy and de-identified. Transcripts were independently analyzed by two members of the research team (IL and JS) using NVivo 14 software (Lumivero). Data interpretation was based on grounded theory methodology, allowing the data itself to guide the conclusions.^22^ Open coding was performed to generate preliminary themes; then a coding structure and code definitions were developed in secondary coding. These were iteratively updated and refined using a constant comparative method.^23^ Inductive analysis continued until thematic saturation was reached. Any disagreements in coding were resolved through discussion and consensus along with a senior author (AF). Themes were finalized and a final codebook was developed iteratively through consensus meetings. Intercoder reliability exceeded 90%. Frequencies of theme occurrence were recorded to illustrate the relative prominence of barriers and facilitators but were not intended to represent quantitative prevalence.

## Results

### Background characteristics

Forty-five individuals participated: 30 patients with foregut cancer and 15 caregivers. The majority of patients were male (77%) and Black (60%). Caregivers were mostly female (87%), Black (73%) and spouses of patients (67%). Cancer diagnoses included pancreatic (50%), gastric/esophageal (27%), and gallbladder cancer/cholangiocarcinoma (23%). Participants represented a spectrum of disease stages and treatment phases. Four patients with metastatic cancer were receiving systemic therapy; the remaining 26 patients included patients with non-metastatic cancer who were receiving or who had completed neoadjuvant chemotherapy (n = 11), those in the postoperative recovery phase (n = 11), and individuals under surveillance (n = 4). Patient and caregiver sociodemographic and access-related variables are reported in Tables 1 and 2.

### Barriers to effective patient-physician communication

Among the 45 participants, 23 explicitly described barriers to communications (Table 3). The most frequently cited barriers included information overload and lack of clarity (65%), lack of clear expectation setting regarding prognosis and treatment impact (52%), lack of acknowledgement of patient or caregiver concerns (17%), and perceived inconsistencies in communication among providers (13%).

#### Information overload and lack of clarity

Patients and caregivers found the use of medical jargon confusing and reported receiving unclear information from their physicians and healthcare teams. A third of participants described lack of clarity and information overload, especially during initial consultations, making it difficult to process and retain key details. One patient reflected: *“.when we first came in was when it was really the toughest because there was a whole lot of information all at one time, and my brain just couldn’t absorb it all.”*. The use of medical jargon and complex medical terminology without sufficient explanations contributed to confusion with a patient stating: *“sometimes when he tells me what’s going on, I don’t exactly understand it in doctor’s terms.”*, and a caregiver emphasizing that “*No one explains anything to you, no one tells, no one explains what or why they are doing things”*.

#### Lack of clear expectation setting regarding prognosis and treatment impact

Several participants reported that their physicians failed to prepare them for the potential outcomes of treatment, particularly around potential complications and side effects, that in some cases led to decisional regret. A patient reflected: *“I would not have had this surgery if I knew that it would be so bad. He told me about the operation but didn’t tell me all the bad things that could happen after. It is now 6 months, 7 months and I still can’t eat.”*. A caregiver similarly expressed: *“It’s just frustrating. Because he didn’t tell us that all these things could happen. I mean, it’s not my job to know. You have to tell us.”*. Another caregiver remarked: “*Not exactly told what to expect, but we’re hoping since he’s been through this before, they can do the same thing again – shrink it down until it is gone.”*, revealing how assumptions may substitute accurate information, when patients and caregivers are not explicitly informed about what treatments entail, or what to expect.

#### Lack of acknowledgement of patient or caregiver concerns

Participants reported instances where providers appeared inattentive to, or minimized their concerns, and where attempts to seek clarification were met with impatience. A patient expressed frustration stating: “*I’m talking to Dr. [Name], and it’s like, my mouth is moving, but she’s not hearing me”*. Another patient described their oncologist as being inattentive: *“He talks yet he doesn’t. It’s like he’ll sit here twiddling his fingers and looking down, but I’ll be really serious about all my questions.”* Caregivers expressed similar feelings of having their loved ones’ symptoms discounted, with one caregiver sharing: “*Dr. [Name] kept telling her things like this is just all in your head, and this went on for a year while she got worse and worse”*. Another caregiver expressed similar sentiments: *“No one explains anything to you, no one tells, no one explains what or why they are doing things… And when you have questions they get impatient.”* These experiences reflect the perceived lack of empathy and connection with their physicians during clinical encounters.

#### Perceived inconsistencies in communication among providers

Participants described receiving inconsistent, and sometimes conflicting information from different physicians, creating confusion and undermining confidence. Discrepancies were reported in communication regarding diagnosis, staging, prognosis, and treatment options, making it difficult for them to fully understand the nature of their disease and at times, eroding trust in the care team. One patient shared: “*They said it wasn’t curable, when Dr. [Name] said it was… you get two different stories from the whole situation now. That’s kind of frustrating”*. Another patient who was previously told that he could not have surgery for his cholangiocarcinoma but was informed that he could during a second opinion over a year later expressed similar confusion, stating: *“After talking with Dr. [Name], I’m kind of disappointed that they didn’t tell me that I did have an option of surgery before now.”*

### Facilitators of effective communication

Facilitators to communication were highlighted by 29 out of the 45 participants of this study (Table 3). The most commonly reported facilitators included clear explanations using accessible language (66%), written or visual materials (55%), feeling heard (48%), opportunities to voice personal goals and concerns (38%), and presentation of treatment options and alternatives (3%).

#### Clear explanations using accessible language

Participants described positive experiences when physicians took the time to explain complex information in simple, understandable language; an approach that reduced anxiety and improved trust. As one patient shared: *“…they cut some of my fear down because they explain everything to me…,”*, while another reflected: *“I really don’t understand doctor talk… but they break it down for me.”*.

#### Written or visual materials

The use of effective written or visual materials was described by participants as helpful to improve their understanding of their cancer care, revisit details, clarify next steps, and feel more confident navigating their treatment journey. One participant noted: *“I wasn’t surprised when I froze my fingertips on ice cubes or my lips got raw. All that was in (the material), that could happen.”*. Visual aids also played a key role, with one patient appreciating that *“they draw diagrams of what he was going to do and how he was going to do it.”* and another comparing the superiority of this approach compared to their past experiences: *“You’ve got some doctors that just tell you, but here they show you…”*.

#### Feeling Heard

Participants expressed appreciation for the physicians who demonstrated active listening and compassion, and treated them as individuals, with one patient noting: “*They listen to you, and then they react, so they don’t just make you feel like you’re just a study or just anybody. They actually listen to you…”*. Caregivers also valued inclusivity during consultations, recognizing when physicians made an effort to include them and understand their perspectives. One caregiver shared: “*I love when Dr. [Name] asks him how he’s feeling, and she looks at him, but then she looks at me to see if I agree with what he’s saying… I appreciate that she knows he is going to give her the answer she wants to hear, and I’m probably going to give the answer that is actually happening”*.

#### Opportunities to voice personal goals and concerns

Participants appreciated physicians who created a safe environment where they were able to voice their concerns, discuss fears and personal goals. This allowed them to align treatments with their personal values, reinstating their active role in the cancer journey. One patient shared: *“I told them my goal is to make it till after the first of the year … I don’t want (my family) to see that holiday as anniversary of something sad…”* highlighting the significance of personal goals in treatment planning. Another noted, *“I’m comfortable asking questions because I know if I don’t speak with them, I won’t be heard.”* reflecting the importance of being empowered to advocate for oneself. A caregiver also affirmed the significance of an open and transparent consultation environment: “*Dr. [Name], he is comfortable talking to her… It’s just like family, and it makes us feel comfortable to say whatever we have to say…*”.

#### Presentation of treatment options and alternatives

While this was less frequently mentioned as a facilitator, participants reported that it built trust when their physicians explained the rationale behind proposed interventions, presented treatment alternatives, and involved them in the decision-making process. As one patient shared: “*They’ve explained what they’re doing, and why they’re doing it, and options that we’re looking into. I’m not going home with any questions about why or what.”*.

## Discussion

This qualitative study explored the perspectives of patients with foregut cancer and their caregivers, identifying key barriers and facilitators to effective communication within a safety-net and tertiary care facility setting. Our findings highlight the importance of communication in patient-centered care and offer actionable strategies to improve patient-centered communication.

Prior research supports the vital role of effective communication, demonstrating that positive physician behaviors including emotional support, clear communication, and confirmation of understanding significantly enhance patient satisfaction.^24^ However, studies also reveal that patient preferences are frequently neither elicited nor clarified, with physicians often assuming that patients’ primary treatment goals are “getting rid of the cancer” without confirming if treatments align with patients’ values and goals.^25^ This assumption may be particularly problematic in foregut cancers, with frequent presence of micrometa-static disease even when imaging suggests localized disease.^26^ In this context, thorough communication regarding the impact of treatments on prognosis and quality of life becomes critical. The interplay of hope and information-seeking is complex; with some patients actively pursuing information to sustain hope, and others avoiding it to preserve optimism and manage fear.^27^

Information overload emerged as a prominent barrier. Patients and caregivers reported difficulty absorbing the volume and complexity of information presented during early consultations. Although physicians may view this front loading as necessary, patients often have no more than one or two appointments prior to treatments including surgical resection, and struggle to process the large volumes of information delivered. The literature suggests that patients’ information needs evolve over time, where they defer to physician expertise and have low interest in detail early in the treatment journey, while becoming more nuanced information-seeking as their lived experience with their disease and treatments increases.^28^ Caregivers’ informational needs, particularly around surgical recovery and discharge are also often over-looked, despite their central role in patients’ care.^29,30^ This underscores the need for a personalized approach that evolves over time, and is responsive to preferred level of involvement and literacy levels.^31^ This includes not only technical details like anatomy and surgical plans, but also integrating psychosocial and self-care information, aspects that are often ignored, despite their importance to patients and caregivers.^30^

Participants also described inadequate expectation setting regarding prognosis, treatment outcomes, and likelihood of cure. This is particularly concerning in foregut surgical oncology, where operations are high-risk and associated with considerable mortality and a not insignificant percentage of ultimately “futile” operations.^32–34^ The use of vague language such as “big surgery” may inadvertently lead patients to conflate treatment with cure.^35^ Omission of detailed prognostic discussions may result in conflation of the goals of treatment with the possibility of cure, fostering unrealistic optimism, distorting informed consent and impairing the alignment of treatment with patient values.^35,36^ Some participants also described decisional regret, stating that they would not have pursued surgery had they understood potential complications and long-term impact on their quality of life. These findings reflect the critical tension between maintaining hope and delivering realistic information, highlighting the need to reframe treatment discussions around achievable goals.^35^ Clear, honest communication is necessary to support informed decision-making but also to reframe hope around symptom control and quality of life. Quantitative studies have shown this communication disconnect, between patient and surgeon understanding of surgical goals and curative potential, even after surgical consultation.^37^

Participants’ accounts of inconsistent or conflicting communication are also notable. While these experiences may reflect the complex realities of modern multidisciplinary care, where patients are evaluated by multiple subspecialists with distinct clinical expertise, they remain consequential. The literature suggests that inconsistent communication may cause confusion, frustration, and feelings of helplessness among patients and caregivers.^38^ Variations in expertise and evolving information are natural features of complex care, assessments of resectability may vary based on subspecialty training and institutional resources,^39^ and second opinions may lead to new recommendations.^40^ However, when this is not clearly communicated, or when visits are rushed or overly technical, patients and caregivers may experience these differences as contraindications, resulting in confusion, and erosion of trust.

Other reported barriers included limited discussion of treatment options, and inability to voice concerns and personal goals. In a study of patients with rectal cancer, Fritz et al. observed that while surgeons presented options and tradeoffs, they rarely clarified patient goals or confirmed understanding, which left patients uncertain about the rationale behind recommendations.^25^ Cultural norms or healthcare dynamics may also discourage patients, particularly in vulnerable populations, from voicing concerns, resulting in a default to a passive “doctor knows best” approach, which may leave preferences unspoken.^41^ Other reasons for hesitancy may include uncertainty about how or whom to approach, or fear of damaging their relationship with providers.^42^ These barriers are likely amplified in safety-net hospitals and other resource-limited settings, and in vulnerable populations, where health literacy may be lower and appointment times more constrained.

Some of these challenges may be reflective of the pressures of today’s clinical environment. Increasing documentation demands, productivity pressures and time constraints, often reinforced by Relative Value Unit (RVU) based reimbursement models, limit opportunities for human-centered interactions.^43^ In the high-stakes context of foregut oncology, these constraints can be particularly detrimental, given the complexity of treatment decisions and the life-altering nature of outcomes. As time and resources continue to grow more constrained, team-based strategies such as nurse navigation and other support personnel may be particularly impactful in reinforcing messages, improving continuity and clarifying information.^44^ Structured tools to assess patient and caregiver health literacy and preferred involvement in shared decision-making could streamline communication while meeting informational needs.^45^ Written and audiovisual materials can reduce cognitive burden, particularly when aligned with literacy level and cultural context.^29^

Taken together, these findings support several actionable strategies to improve communication. Communication training could help physicians prioritize active listening, avoid medical jargon, use plain language and routinely confirm patient understanding. Transparency regarding the benefits, limitations and likely impact of treatment on quality of life is essential, as is consistency in messaging across the multidisciplinary team. Providing patients and caregivers with written materials, visual aids, and time and space to articulate their concerns and goals may improve alignment between patient values and treatment plans. Importantly, many of these interventions are practical and scalable even in resource-constrained settings, particularly when embedded within team-based workflows and supported by standardized educational tools.

One of the most complex tasks in foregut oncology is balancing hope with realism - a goal best achieved through thoughtful, honest, patient-centered communication. Clarity should not be mistaken for pessimism, but rather an opportunity to reframe hope around achievable, specific goals including symptom relief, functional preservation, and quality of life, alongside or in place of a cure.^35^ A structured strategy such as the Best Case/Worst Case framework, that provides a visual and narrative representation of potential scenarios may be particularly valuable in this context. By outlining the best, worst and most likely outcomes, physicians can discuss treatment options, clarify tradeoffs, and support patients and caregivers in setting realistic expectations, while ensuring that decisions are aligned with patients’ values, goals, and quality-of-life priorities.^46^

While this study provides valuable insights, several limitations need to be acknowledged. The study was conducted at a single center in the Southeastern US, and this limits its generalizability. Therefore, while our study setting provides crucial insights into the perspectives of an underserved population, these findings may not reflect the experiences of patients and caregivers in other geographical and socioeconomic settings. Additionally, given that the interviews were not designed exclusively to assess quality of communications, participants were not robustly prompted to communicate all possible barriers and facilitators to effective communication. Although participants were interviewed across different phases of care, interviews were conducted at a single time point and may not fully capture how communication needs evolve longitudinally across the cancer care continuum.

## Conclusion

This study underscores communication as a critical, modifiable determinant of patient and caregiver experience in foregut cancer care. Structured, patient-centered strategies that prioritize clarity, consistency, empathy, and are tailored to individual health literacy levels, can foster trust and support shared decision-making. Incorporating team-based approaches may help reinforce key messages. Future efforts should focus on incorporating diverse stakeholder perspectives to develop educational toolkits that assess health literacy, communication, and decision-making preferences, thus allowing clinicians to individualize care while ensuring patients and caregivers are informed, heard, and meaningfully engaged.

## Supplementary Material

1

2

## Figures and Tables

**Table 1:** Patient sociodemographic and access related characteristics.

Characteristics	N = 30
**Sociodemographic Characteristics**	
**Age,** median (IQR)	63 (55, 67) years
**Race,** n (%)	
Black	18 (60%)
White	12 (40%)
**Gender,** n (%)	
Female	7 (23%)
Male	23 (77%)
**Insurance,** n (%)	
Private	6 (20%)
Medicare	11 (37%)
Medicaid	3 (10%)
VA	3 (10%)
Uninsured	7 (23%)
**Area Deprivation,** n (%)	
Low	4 (13%)
Intermediate	7 (23%)
High	19 (63%)
**ECOG,** n (%)	
0–1	21 (70%)
≥ 2	9 (30%)
**Cancer type,** n (%)	
Pancreatic	15 (50%)
Gastric - Esophageal	8 (27%)
Cholangiocarcinoma - Gallbladder	7 (23%)
**Access Related Characteristics**	
**Receipt of Guideline Concordant Treatment,** n (%)	15 (50%)
**Presence of PCP,** n (%)	18 (60%)
**Time from Symptom Onset to Diagnostic Work-Up,** median (IQR)	9.7 (6.1, 15.6) weeks
**Time from Diagnosis to Oncology Appointment,** median (IQR)	5.9 (4.7, 7.3) weeks
**Time from Oncology Appointment to Treatment,** median (IQR)	5.0 (3.4, 6.1) weeks

**Table 2:** Caregiver sociodemographic characteristics and their patients’ context.

Characteristic	N = 15
**Age**, median (IQR)	63 (49, 68)
**Gender**, n (%)	
Female	13 (87%)
Male	2 (13%)
**Race**, n (%)	
White	3 (20%)
Black	11 (73%)
Other	1 (6.7%)
**Area Deprivation**, n (%)	
Low	1 (6.7%)
Intermediate	3 (20%)
High	11 (73%)
**Relationship to the patient**, n (%)	
Spouse	10 (67%)
Child	3 (20%)
Parent	2 (13%)
**Patient Insurance**, n (%)	
Private	4 (27%)
Medicare	5 (33%)
Medicaid	5 (33%)
Self-pay	1 (6.7%)
**Patient Cancer**, n (%)	
Pancreatic	7 (47%)
Esophageal	2 (13%)
Gastric - GEJ	4 (27%)
Cholangiocarcinoma - Gallbladder	2 (13%)

**Table 3:** Patients’ and caregivers’ reported barriers and facilitators to effective communication.

Category	Theme	Quote
Barriers to Effective Communication	**Information Overload And Lack Of Clarity**	*“…when we first came in was when it was really the toughest because there was a whole lot of information all at one time, and my brain just couldn’t absorb it all.” (Patient)* *“No one explains anything to you, no one tells, no one explains what or why they are doing things… they just tell you what to do but no one explains. And when you have questions they get impatient.” (Caregiver)*
	**Lack Of Clear Expectation Setting Regarding Prognosis And Treatment Impact**	*I would not have had this surgery if I knew that it would be so bad. He told me about the operation but didn’t tell me all the bad things that could happen after. It is now 6 months, 7 months and I still can’t eat. (Patient – Caregiver Interview)* *I’m talking to her, and it’s like, my mouth is moving, but she’s not hearing me. She’s got a job to do, and I know that, but our personalities just clash a little bit. (Patient)*
	**Lack Of Acknowledgement Of Patient Or Caregiver Concerns**	*“I’m talking to her, and it’s like, my mouth is moving, but she’s not hearing me. She’s got a job to do, and I know that, but our personalities just clash a little bit” (Patient)* *“[Doctor] kept telling things like this is just all in your head, and this went on for a year while she got worse and worse…” (Caregiver)*
	**Perceived Inconsistencies In Communication Among Providers.**	*“Some of my doctors said that it was a good chance it [chemotherapy] would get rid of it all. And some of them said, you’re still going to have to have surgery. I was really confused.” (Patient)*
Facilitators to Effective Communication	**Clear Explanations Using Accessible Language**	*“The doctors have been treating me really well, and they’ve been upfront with me, telling me everything and drawing of what they were going to do, and how they were going to do it…” (Patient)* *“To me it was like going to school. We are getting a blessing, you telling us about the different things*… *and it was like an educational kind of thing. So that’s helpful.” (Caregiver)*
	**Written Or Visual Materials**	*“I wasn’t surprised when I froze my fingertips on ice cubes or my lips got raw. All that was in (the material), that that could happen.” (Patient)* *“We do (receive written material) when we leave the hospital. If I were to ask like, can you give me a diet? She would print something off for me…” (Caregiver)*
	**Feeling Heard**	*“… so they don’t just make you feel like you’re just a study or just anybody. They actually listen to you, and they react off what you tell them. A lot of places don’t do that.” (Patient)* *“… I appreciate that she knows he is going to give her the answer she wants to hear, and I’m probably going to give the answer that is actually happening.” (Caregiver)*
	**Opportunities To Voice Personal Goals And Concerns**	*“Dr. [Name], he is comfortable talking to her*… *It’s just like family, and it makes us feel comfortable to say whatever we have to say.” (Caregiver)*
	**Presentation Of Treatment Options And Alternatives.**	*“They’ve explained what they’re doing, and why they’re doing it, and options that we’re looking into. I’m not going home with any questions about why or what” (Patient)*

## Appendix A. Supporting information

Supplementary data associated with this article can be found in the online version at doi:10.1016/j.soi.2026.100238.
