# Supplementary material for: Bridging the communication gap in foregut cancer: A qualitative exploration of patient and caregiver perspectives
Source: Surg Oncol Insight. Author manuscript; Available in PMC 2026 Mar 26. (PMC13015860; doi:10.1016/j.soi.2026.100238)
Supplement: 2 [file NIHMS2156261-supplement-2.pdf]

Barriers to Oncologic Care Interview Guide

**I'd like to talk about your experience with your cancer treatment.**
1. How has your experience with the medical system been, from the time you started having symptoms of cancer until now?
2. How satisfied are you with the medical care you received after your cancer diagnosis?<br><br>IF NOT MENTIONED:<br>☐ What makes you say satisfied/unsatisfied [depending on prior response]?
3. About how long was it from the time your symptoms started to when you received your cancer diagnosis?<br><br>4. How satisfied are you with the care you received from the time your symptoms started to when you received your cancer diagnosis?<br><br>IF NOT MENTIONED:<br>☐ What makes you say satisfied/unsatisfied [depending on prior response]?
5. What makes it hard to follow up on your cancer treatment? (*Alternative wording:* What makes it hard for you to get treatment?)<br><br>IF PATIENT SEEMS UNSURE:<br>☐ Other people have said that some things make it hard for them to get their cancer treatment. Sometimes people have trouble being able to get an appointment to see a cancer doctor, getting to doctor's appointments, following doctors' recommendations, or getting medications. Has anything like that been a problem for you?<br><br>☐ You said __________ [list what they identified]. Can you tell me more about that?<br><br>☐ How are you dealing with this?<br><br>☐ Where are you getting help from?

Next, I am going to ask about some of the things other people have said make it harder or easier to follow up with cancer treatment. (ONLY ASK ABOUT THINGS THAT THEY DIDN'T MENTION)

a) How helpful is your cancer care team when issues come up?

IF PATIENT SEEMS UNSURE: By cancer care team I mean all the people who help treat your cancer both medically and surgically

- ☐ Can you tell me more about that? *If helpful:* What do they do that is helpful?
- ☐ *If not helpful:* Can you tell me more about that?
- ☐ *If not helpful:* How are you dealing with this? Where are you getting help from?

b) Do you feel like your cancer doctors and cancer care team listens to you?

- ☐ *If no:* Can you tell me more about that?
- ☐ *If no:* How are you dealing with this? Where are you getting help from?

c) Do you have problems with getting to and from your appointments? (Examples include: issues with travel distance)

- ☐ *If yes:* Can you tell me more about that?
- ☐ *If yes:* How are you dealing with this? Where are you getting help from?

Follow up: how long does it typically take you to get to your appointment?

d) Do you have problems with treatment costs or payment? (Examples include: co-pays, cost of transportation, loss of wages for you or for family members)

- ☐ *If yes:* Can you tell me more about that?
- ☐ *If yes:* How are you dealing with this? Where are you getting help from?

Follow up: Do you have insurance? If no: Has this been an issue?

e) Do you have problems with how long your doctor's appointments usually take?

- *If yes:* Can you tell me more about that?
- *If yes:* How are you dealing with this? Where are you getting help from?

FOR LONG APPOINTMENTS:

- How do you plan for the timing for a doctor's appointment?
- How do you use that time at the doctor's office?

IF NOT MENTIONED:

- How do you feel before the appointment? How do you feel afterward?

f) Do you have problems understanding your doctors' instructions?

- *If yes:* Can you tell me more about that?
- *If yes:* How are you dealing with this? Where are you getting help from?

g) Do you have problems remembering your doctors' instructions?

- *If yes:* Can you tell me more about that?
- *If yes:* How are you dealing with this? Where are you getting help from?

h) How much help do you have from family or friends?

- Can you tell me more about that? *If helpful:* What do they do that is helpful?
- *If not helpful:* Can you tell me more about that?
- *If not helpful:* How are you dealing with this? Where are you getting help from?

i) Do you have difficulties dealing with worry, anxiety, or sadness?

- *If yes:* Can you tell me more about that?
- *If yes:* How are you dealing with this? Where are you getting help from?

j) Do you have problems with discrimination?

- *If yes:* Can you tell me more about that?
- *If yes:* How are you dealing with this? Where are you getting help from?

k) Do you have problems with pain?

- *If yes:* Can you tell me more about that?
- *If yes:* How are you dealing with this? Where are you getting help from?

l) Do you have problems getting and taking your medications?

- *If yes:* Can you tell me more about that?
- *If yes:* How are you dealing with this? Where are you getting help from?

m) Do you have problems with treatment side effects?

IF SIDE EFFECTS ARE A PROBLEM:

- Can you tell me more about that?
- Have you called your doctor's office to follow up on a side effect?
- *If yes:* How did you feel before the phone call? How did you feel after you received a response?
- *If no:* What made you not call the doctor's office to follow up on a side effect?

FOR ALL RESPONDENTS:

- Did your doctor talk to you about the side-effects that you should expect before starting treatment?
- *If yes*: How was that discussion? Did you understand what was explained to you?

- *If yes:* Would you change anything about that discussion you had with your doctor?

6. In general, how do you get medical advice in between appointments if a problem comes up? (*Note: Assess ease of access, smoothness of process, etc.)*

   - What would you like to change about how you can get medical advice?

7. How helpful is the hospital at addressing things that make it harder for you to get cancer treatment?

   IF NOT MENTIONED:
   - How is the hospital helping?
   - How would you like them to help?
   - What could the hospital do to help you more?

Next, I am going to ask you questions about your doctor(s) and care team.

8. How is your relationship with your cancer doctors and cancer care teams?
   IF PATIENT SEEMS UNSURE: By cancer care team I mean all the people who help treat your cancer both medically and surgically

   - Can you tell me more about it?

9. How much trust do you have for the medical system?

   - Can you tell me more about it?

10. How much trust do you have for your cancer doctors?

    - Can you tell me more about it?

11. How comfortable are you asking them questions and telling them your concerns?

☐ Can you tell me more about it?

If no: what would make you more comfortable?

12. Do you have a primary care physician?

☐ *If yes:* What could your primary care team do to help you more?

☐ *If no:* Would you like to establish care with a primary care physician? (IF YES: What help would you need to make that happen?)

I'd like to talk about what helps with your cancer treatment.

13. In general, what currently helps your cancer treatment? This could be anything

☐ Can you tell me more about that?

Next, I am going to ask about some of the things other people have said help them through their cancer treatment. (ONLY ASK ABOUT THINGS THAT THEY DIDN'T ALREADY MENTION)

a) Do you have support from family or friends?

☐ *If yes:* How helpful has this been for you?

☐ *If no:* How helpful would this be for you?

b) Do you attend support groups?

☐ *If yes:* How helpful has this been for you?

☐ *If no:* How helpful would this be for you?

c) Have you talked to other cancer survivors?

☐ *If yes:* How helpful has this been for you?

- ☐ *If no:* How helpful would this be for you?

d) Have you talked with a counselor?

- ☐ *If yes:* How helpful has this been for you?
- ☐ *If no:* How helpful would this be for you?

e) Do you receive help from a social worker?

- ☐ *If yes:* How helpful has this been for you?
- ☐ *If no:* How helpful would this be for you?

f) Do you receive help from a patient care advocate? (*if necessary, say*: When I saw patient care advocate, I mean a professional who could help coordinate your care, connect you with resources, and advocate for your healthcare needs?)

- ☐ *If yes:* How helpful has this been for you?
- ☐ *If no:* How helpful would this be for you?

g) Do you have a relationship with your medical providers?

- ☐ *If yes:* How helpful has this been for you?
- ☐ *If no:* How helpful would this be for you?

h) Do you receive help dealing with worry or sadness?

- ☐ *If yes:* How helpful has this been for you?
- ☐ *If no:* How helpful would this be for you?

i) Have you talked through your cancer treatment goals with your cancer care team?

☐ *If yes:* How helpful has this been for you?

☐ *If no:* How helpful would this be for you?

j) Have you been told what to expect from your cancer treatment?

☐ *If yes:* How helpful has this been for you?

☐ *If no:* How helpful would this be for you?

k) Do you receive help with transportation?

☐ *If yes:* How helpful has this been for you?

☐ *If no:* How helpful would this be for you?

l) How helpful is reading information provided to you by your doctor? For example, has your doctor given you any information to read?

☐ *If yes:* How helpful has this been for you?

☐ *If no:* How helpful would this be for you?

m) Have you used any online resources or information?

☐ *If yes:* How helpful has this been for you?

☐ *If no:* How helpful would this be for you?

n) Do you receive written, electronic or other instructions and reminders about tests and appointments?

☐ *If yes:* How helpful has this been for you?

☐ *If no:* How helpful would this be for you?
o) Do you identify as spiritual or religious?<br><br>☐ *If yes:* How helpful is your faith or beliefs in getting you through your cancer treatment?<br>☐ *If no:* How helpful would this be for you?
p) Have you talked to someone about end-of-life care?<br><br>☐ *If yes:* How helpful has this been for you? How does talking about it make you feel?<br><br>☐ *If no:* How helpful would this be for you?
14. What other things do you think would help <u>you</u> as you go through your cancer treatment?
15. What other things could be done to improve your overall cancer treatment?
16. What would you change about the process of calling your doctor's office?
17. What advice would you give to someone else trying to receive cancer treatment?
18. What else do you think would be helpful for us to know so that we can help others with their cancer treatment?

Thank you for your time and answers to these questions.

Barriers to Oncologic Care Interview Guide (Caregiver Protocol)

1. Overall, how has your experience been since your ___ was diagnosed with cancer?

    IF NOT MENTIONED:

    - What makes you say satisfied/unsatisfied [depending on prior response]?

2. Was this diagnosed because of symptoms that s/he had?

3. How satisfied are you with the care your _____received after his/her symptoms started…or from the time symptoms started till they received his/her cancer diagnosis?

    IF NOT MENTIONED:

    - What makes you say satisfied/unsatisfied [depending on prior response]?

4. Are there things that make it difficult for him/her/they to get treatment?

    IF PATIENT SEEMS UNSURE:

    - Other people have said that some things make it hard for them to get their cancer treatment. Sometimes people have trouble being able to get an appointment to see a cancer doctor, getting to doctor's appointments, following doctors' recommendations, or getting medications. Has anything like that been a problem for you?
    - You said __________ [list what they identified]. Can you tell me more about that?
    - How are you dealing with this?
    - Where are you getting help from?

Thank you for taking the time to speak with me about this important topic. I'd like to discuss your experience since your ___ was diagnosed with cancer.

5. How are you handling/coping with their cancer diagnosis?

- Can you tell me more about that?

6. Are there any problems overall with his/her cancer treatment?

- Can you tell me more about that?

Next, I am going to ask about some of the things other people have said make it harder or easier for them as their loved ones get cancer treatment(ONLY ASK ABOUT THINGS THAT THEY DIDN'T MENTION)

a) Do you feel you have help from his/her cancer care team?

- Can you tell me more about that? *If helpful:* What do they do that is helpful?
- *If not helpful:* Can you tell me more about that?
- *If not helpful:* How are you dealing with this? Where are you getting help from?

b) Do you feel like the cancer care team listens to your

concerns? - *If no:* Can you tell me more about that?

- *If no:* How are you dealing with this? Where are you getting help from?

c) Do you have problems with getting to and from your appointments? (Examples include: issues with travel distance)

- *If yes:* Can you tell me more about that?

- *If yes:* How are you dealing with this? Where are you getting help from?

d) Do you have problems with treatment costs or payment? (Examples include: co-pays, cost of transportation, loss of wages for you or for family members)

Follow up question : Is your _____ insured?

- *If yes:* Can you tell me more about that?

- *If yes:* How are you dealing with this? Where are you getting help from?

e) Do you have problems understanding his/her doctors'

instructions? *If yes:* Can you tell me more about that?

- *If yes:* How are you dealing with this? Where are you getting help from?

f) Do you have problems remembering your doctors' instructions? ■ *If yes:* Can you tell me more about that?

■ *If yes:* How are you dealing with this? Where are you getting help from?

g) Do you feel like you have enough support from family or friends? ■ Can you tell me more about that? *If helpful:* What do they do that is helpful?

■ *If not helpful:* Can you tell me more about that?

■ *If not helpful:* How are you dealing with this? Where are you getting help from?

h) Do you have difficulties dealing with worry, anxiety, or sadness?

■ *If yes:* Can you tell me more about that?

■ *If yes:* How are you dealing with this? Where are you getting help from?

i) Have you experienced feelings of inequality?<br><br>- Definition *:* Uneven or unfair distribution of opportunities, benefits,and treatment<br>- *If yes:* Can you tell me more about that?<br>- *If yes:* How are you dealing with this? Where are you getting help from?
j) How do you get medical advice between appointments if something comes up?<br><br>- *What would you like to change about how you can get medical advice?*<br><br>k) Do you feel you are able to help your ___ sufficiently?<br><br>- *If yes:* Can you tell me more about that?<br>- *If no:* How are you handling/coping with this?
l) Is there anything that the hospital or your cancer team can do to help you?<br><br>- Can you tell me more about that?
7. In general, how do you get medical advice in between appointments if a problem comes up? (*Note: Assess ease of access, smoothness of process, etc.)*<br><br>- What would you like to change about how you can get medical advice?

Next, I am going to ask you questions about your doctor(s) and care team.

8. How is your relationship with your ______ cancer doctors and cancer care team?

- Can you tell me more about it?

9. How much trust do you have for the medical system?

- Use 1-5 scale if patient is hesitant to respond

  1 = Little trust

  5 = A lot of trust

- Can you tell me more about it?

10. Do you trust his/her specific cancer doctors?

- Use 1-5 scale if patient is hesitant to respond

  1 = Little trust

  5 = A lot of trust

- Can you tell me more about it?

11. Are you comfortable asking them questions and telling them your concerns?

- Can you tell me more about it?

12. Do you feel they listen and help with your concerns?

- *If yes:* Can you tell me more about this?
- *If no:* What could your care team do to help you more?

I am going to ask about some of the things other people have said help them as their loved ones go through cancer treatment (ONLY ASK ABOUT THINGS THAT THEY DIDN'T ALREADY MENTION)

a) Do you have support from family or friends?

- *If yes:* How helpful has this been for you?
- *If no:* How helpful would this be for you?

b) Do you attend support groups?

- *If yes:* How helpful has this been for you?
- *If no:* How helpful would this be for you?

c) Would it help to talk to other caregivers of cancer patients?

- *If yes:* How helpful has this been for you?
- *If no:* How helpful would this be for you?

d) Have you talked with a counselor?

- *If yes:* How helpful has this been for you?
- *If no:* How helpful would this be for you?

e) Do you receive help from a social worker?

- *If yes:* How helpful has this been for you?
- *If no:* How helpful would this be for you?

f) Do you identify as spiritual or religious?

- *If yes:* How helpful is your faith or beliefs in getting you through your cancer treatment?
- *If no:* How helpful would this be for you?

g) Do you receive help dealing with worry or sadness?<br><br>*If yes:* How helpful has this been for you?<br><br>*If no:* How helpful would this be for you?
h) Have you been told what to expect from your _____ cancer treatment? *If yes:* How helpful has this been for you?<br><br>*If no:* How helpful would this be for you?
i) Would it help to get reading information from your doctor? Has your ______doctor/s given you any information to read?<br><br>*If yes:* How helpful has this been for you?<br><br>*If no:* How helpful would this be for you?
j) Do you receive written instructions and reminders about your ______tests and appointments?<br><br>*If yes:* How helpful has this been for you?<br><br>*If no:* How helpful would this be for you?
13. If you could wave a magical wand, what could the hospital do to help your ______ through their cancer care journey?<br><br>Can you tell me more about it?

14. What advice would you give to other caregivers of cancer patients?

15. What else do you think would be helpful for us to know so that we can help other caregivers in similar situations?

Thank you for your time and answers to these questions.
